# Is it time to reduce the length of postgraduate training for physician-scientists in internal medicine?

**DOI:** 10.1172/jci.insight.178214

**Published:** 2024-05-22

**Authors:** Emily Jane Gallagher, Paul R. Conlin, Barbara I. Kazmierczak, Jatin M. Vyas, Olujimi A. Ajijola, Christopher D. Kontos, Robert A. Baiocchi, Kyu Y. Rhee, Patrick J. Hu, Carlos M. Isales, Christopher S. Williams, Don C. Rockey

**Affiliations:** 1 Division of Endocrinology, Diabetes and Bone Disease, Department of Medicine, Icahn School of Medicine at Mount Sinai, New York, New York, USA.; 2 VA Boston Healthcare System and Harvard Medical School, Boston, Massachusetts, USA.; 3 Department of Microbial Pathogenesis, Department of Medicine (Infectious Diseases), Yale University School of Medicine, New Haven, Connecticut, USA.; 4 Division of Infectious Disease, Department of Medicine, Massachusetts General Hospital, Boston, Massachusetts, USA. Department of Medicine, Harvard Medical School, Boston, Massachusetts, USA.; 5 UCLA Cardiac Arrhythmia Center, David Geffen School of Medicine at UCLA, Los Angeles, California, USA.; 6 Department of Medicine, Duke University Medical Center, Durham, North Carolina, USA.; 7 Division of Hematology, Department of Internal Medicine, Comprehensive Cancer Center, The Ohio State University, Columbus, Ohio, USA.; 8 Division of Infectious Diseases, Department of Medicine, Weill Cornell Medicine, New York, New York, USA.; 9 Departments of Medicine and Cell and Developmental Biology, Vanderbilt University Medical Center, Nashville, Tennessee, USA.; 10 Departments of Medicine, Neuroscience and Regenerative Medicine, Medical College of Georgia at Augusta University, Augusta, Georgia, USA.; 11 Department of Medicine, Division of Gastroenterology, Vanderbilt University Medical Center, Nashville, Tennessee, USA.; 12 Veterans Affairs Tennessee Valley Health Care System, Nashville, Tennessee, USA.; 13 Vanderbilt Ingram Cancer Center, Nashville, Tennessee, USA.; 14 Division of Gastroenterology and Hepatology and Digestive Disease Research Center, Medical University of South Carolina, Charleston, South Carolina, USA.

## Abstract

Physician-scientists play a crucial role in advancing medical knowledge and patient care, yet the long periods of time required to complete training may impede expansion of this workforce. We examined the relationship between postgraduate training and time to receipt of NIH or Veterans Affairs career development awards (CDAs) for physician-scientists in internal medicine. Data from NIH RePORTER were analyzed for internal medicine residency graduates who received specific CDAs (K08, K23, K99, or IK2) in 2022. Additionally, information on degrees and training duration was collected. Internal medicine residency graduates constituted 19% of K awardees and 28% of IK2 awardees. Of MD-PhD internal medicine–trained graduates who received a K award, 92% received a K08 award; of MD-only graduates who received a K award, a majority received a K23 award. The median time from medical school graduation to CDA was 9.6 years for K awardees and 10.2 years for IK2 awardees. The time from medical school graduation to K or IK2 award was shorter for US MD-PhD graduates than US MD-only graduates. We propose that the time from medical school graduation to receipt of CDAs must be shortened to accelerate training and retention of physician-scientists.

## Abstract 2

We propose that the time from medical school graduation to receipt of CDAs must be<br />shortened to accelerate training and retention of physician-scientists.

## Introduction

Physician-scientists are a critical component of the biomedical research workforce, advancing medical knowledge and improving patient care through their research. Training physician-scientists requires substantial investment of time and financial resources. Candidates may be discouraged from pursuing this career path due to the enormous commitments and the pressures of competing priorities. With regard to the time commitment, the 2014 NIH Physician-Scientist Working Group reported that the average time from medical school graduation to first independent research award (R01 or equivalent) for individuals with MD-PhD degrees was 13 years and for those with MD-only degrees was 17 years. In 2020, the mean age for recipients obtaining their first R01 was 46 years for both MD and MD-PhD graduates and has steadily increased over the past 30 years (1). Thus, approaches to shorten physician-scientist training duration have taken on increased emphasis.

Recent studies have explored the causes of the lengthening time to obtain an independent research award, focusing primarily on the years before graduating from medical school. The age at matriculation into MD-PhD programs has risen, and the length of time spent in MD-PhD programs has also increased (2, 3). Additionally, it appears that the time spent in postgraduate training (residency and/or fellowship) is increasing.

Career development awards (CDAs), such as NIH K, Veterans Administration (VA) IK2, or foundation awards, support junior faculty for up to 5 years, with protected time to develop independent research careers, and they are often a first step to independent funding for physician-scientists. While most of the studies examining the duration of career development have focused on MD-PhD graduates, it is worth noting that approximately 15% of all individuals receiving their first independent NIH award (R01 or equivalent) are MD-only degree holders, and 10% are MD-PhD holders (4), suggesting that those who develop an interest in research during clinical training make up a sizable proportion of physician-scientists.

We hypothesized that postgraduate training requirements and/or structural factors delay the time to independent research funding for physician-scientists. Therefore, we explored the time from medical school graduation to receipt of CDAs for internal medicine physician-scientists to better understand if this may represent a future target for intervention.

## Results

### K awards types, NIH institutes, degree, and sex.

**Figure 1:**
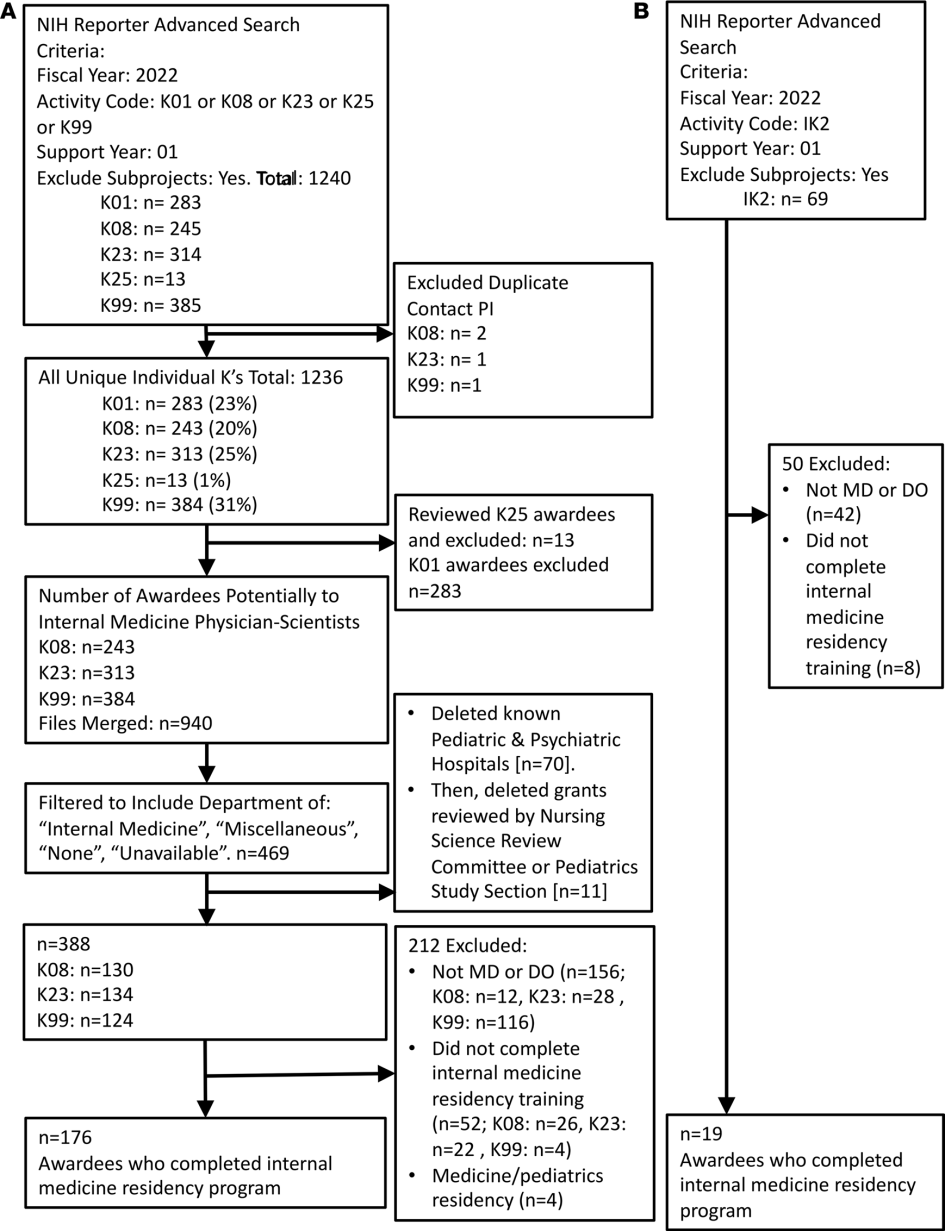
Flow chart summarizing the workflow and exclusion criteria for the study. (A) Flow chart summarizing the NIH RePORTER advanced search results for K01, K08, K23, K25, and K99 awards. (B) Flow chart summarizing the NIH RePORTER advanced search results for IK2 awards.

For fiscal year 2022, we identified 1,236 unique K01, K08, K23, K25, and K99 awardees in their first year of NIH support, of whom 283 (23%) received K01s, 243 (20%) received K08s, 313 (25%) received K23s, 13 (1%) received K25s, and 384 (31%) received K99s (Supplemental Table 1; supplemental material available online with this article; https://doi.org/10.1172/jci.insight.178214DS1). Of these awardees, 176 (14% of the total) were graduates of internal medicine residency programs (Figure 1), with 91 (52%) K08 awardees, 81 (46%) K23 awardees, and 4 (2%) K99 awardees (Table 1). Administering NIH institutes that awarded the greatest proportion of K awards to internal medicine residency graduates included the National Heart, Lung, and Blood Institute (*n* = 58 of 176, 33%), National Institute of Diabetes and Digestive and Kidney Diseases (*n* = 40 of 176, 23%), and National Cancer Institute (NCI) (*n* = 21 of 176, 12%). These 3 NIH institutes were among the top 4 institutes in terms of overall K awards in 2022, although National Institute on Aging, which awarded the second highest number of Ks overall in 2022, granted a very high proportion of K01 awards (56 of 122, 46%; Supplemental Table 1). Notably, in 2022, NCI no longer utilized a K23 award mechanism, so all K awards to internal medicine residency graduates from NCI were K08s.

**Figure 2:**
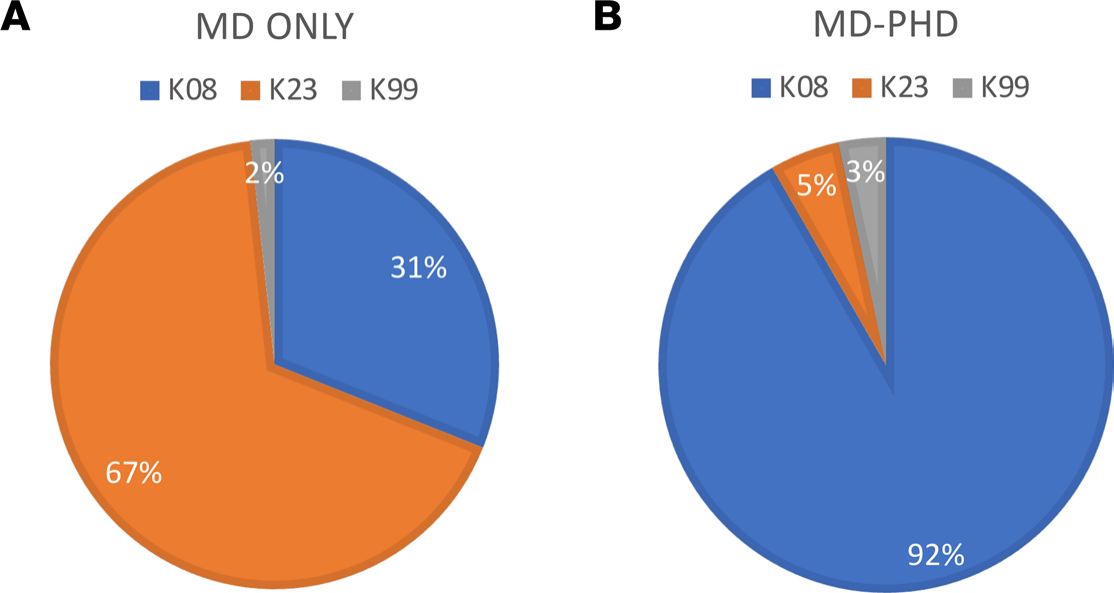
K award type based on degree. (**A**) MD-only graduates who completed internal medicine training (*n* = 116). (**B**) MD-PhD graduates who completed internal medicine residency training (*n* = 60).

Of the K awardees who were graduates from internal medicine residency programs, more were MD-only degree holders (*n* = 116 of 176, 66%) than MD-PhD degree holders (*n* = 60 of 176, 34%). Of those with MD-PhD degrees, a majority were graduates of US MD-PhD programs (*n* = 49 of 60, 82%), and the remainder received their PhD degree after graduating from medical school (*n* = 11 of 60, 18%). No K awardee who graduated from an internal medicine residency program had a Doctor of Osteopathic Medicine (DO) degree. There were substantial differences in the types of K awards held by degree types (Figure 2, A and B). Of MD-PhD graduates, 55 of 60 (92%) received K08 awards followed by K23 (*n* = 3 of 60, 5%) and K99 (*n* = 2 of 60, 3%) awards, while 36 of 116 (31%) MD-only degree holders had K08 awards, 78 of 116 (67%) had K23 awards, and 2 of 116 (2%) had K99 awards.

Ninety of the 176 internal medicine residency graduates with K awards were women (51%). Of MD-PhD graduates, 26 of 60 (43%) were women, while for MD-only graduates, 64 of 116 (55%) were women. Within K award type, women made up 43 of 91 (48%) of K08 awardees, 45 of 81 (56%) of K23 awardees, and 2 of 5 (50%) of K99 awardees.

### Subspecialty training for K awardees.

Of the K awardees, nearly all (98%) of the MD-PhD graduates underwent subspecialty fellowship training, while 91% of MD-only degree holders underwent subspecialty fellowship training. The most common subspecialty for MD-PhD K awardees was hematology/oncology (*n* = 12 of 60, 20%); for MD-only degree holders, it was gastroenterology (*n* = 20 of 116, 17%) (Table 2 and Table 3). Within the subspecialties that had 10 or more K awardees, hematology/oncology and cardiology had the lowest proportion of women (9 of 23, 40% in hematology/oncology, and 10 of 25, 40% in cardiology), followed by infectious diseases (9 of 21, 43% women) and pulmonary critical care (12 of 26, 46% women). Endocrinology had the highest proportion of female K awardees (9 of 11, 82%), followed by rheumatology (7 of 10, 70%), nephrology (11 of 18, 61%), and gastroenterology (13 of 22, 59%).

### US and non-US medical school graduates.

Twenty-eight (16%) of 176 K awardees were graduates of non-US medical schools (K08, *n* = 16; K23, *n* = 10; K99, *n* = 2). Of non-US medical school graduates, 20 of 28 (71%) were MD-only degree holders, and 8 of 28 (29%) were MD-PhD holders. This was not different from the distribution of K awards by degrees among US medical school graduates, where 96 of 148 (65%) were MD only degree holders, and 52 of 148 (35%) were MD-PhD graduates. Of note, all of the non-US MD-PhD degree holders received their PhD degrees after medical school (median, 5 years after medical school graduation; range, 3–7 years). However, only 3 of 52 (6%) MD-PhD holders who were graduates of US medical schools did not graduate from MD-PhD programs, and all of these graduates received their PhD degrees after medical school (range, 7–12 years after medical school graduation).

### IK2 awards: degree, sex, subspecialty, and medical school location.

Of the 19 IK2 awardees who completed internal medicine residency training, 5 (26%) had MD-PhD degrees, 13 (68%) had MD-only degrees, and 1 (5%) had a DO-only degree. All IK2 awardees with MD-PhD degrees were graduates of US MD-PhD programs. All awardees completed subspecialty training, with cardiology being the most common subspecialty (5 of 19, 26%). One IK2 awardee graduated from a non-US medical school. Nine of 19 IK2 awardees (47%) were women.

### Time from medical school graduation to receipt of CDA.

The median time from medical school graduation to K award was 9.6 years (range, 4.2–27.2 years). Among non-US medical school graduates, we hypothesized that some of this lag was driven by training after medical school in other countries or prior to entering a US residency program as well as immigration requirements for NIH CDAs eligibility. Indeed, we found that for non-US medical school graduates the median time from medical school graduation to K award was 15.3 years (range, 8.8–27.2 years), whereas for US medical school graduates it was 9.1 years (range, 4.2–16.8 years) (*P* < 0.001). However, there was no substantial difference in median time from medical school graduation to IK2 award if the non-US medical school graduate was included (10.2 years; range, 5.9–24.7 years) or excluded from the analysis (10.0 years; range, 5.9–12.8 years).

**Figure 3:**
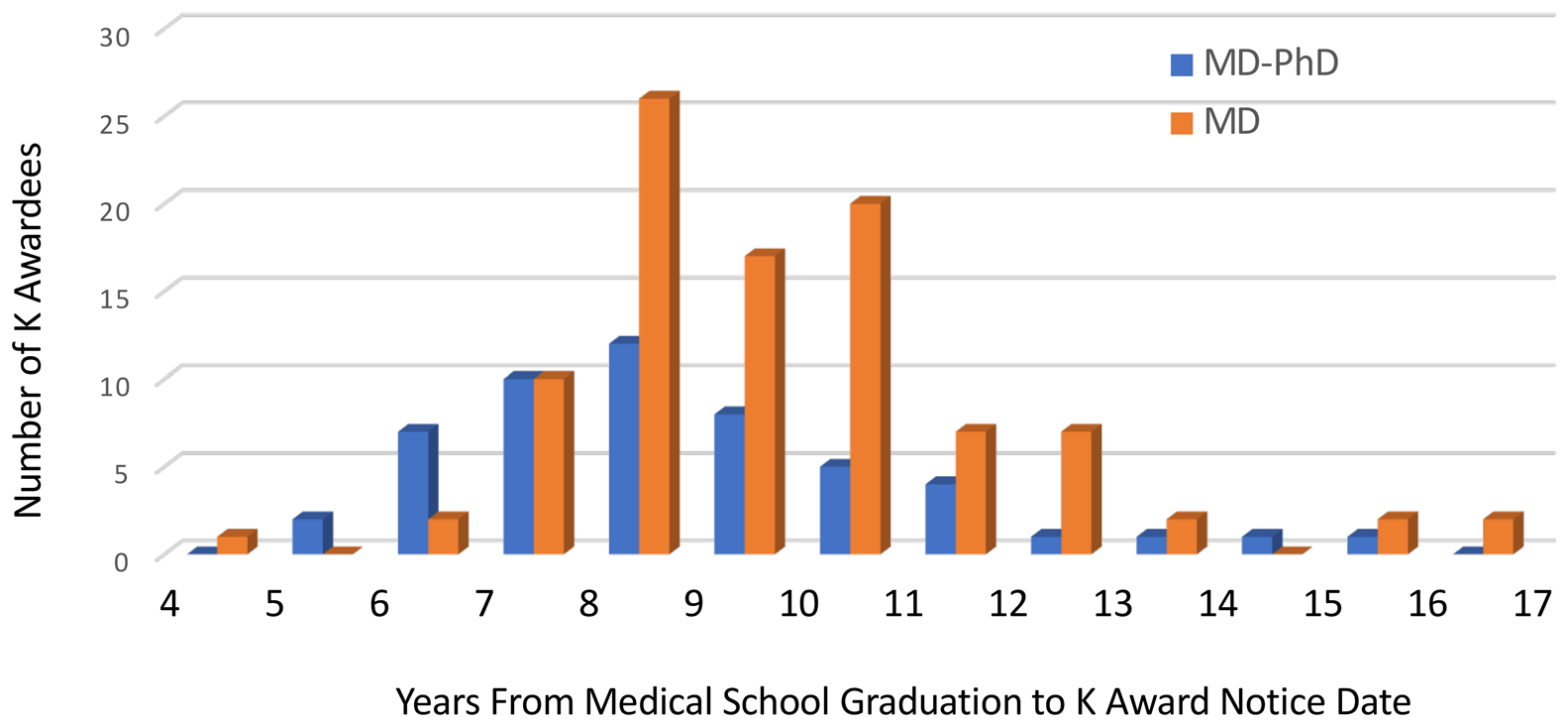
Time from medical school graduation to K award. Histogram comparing years from medical school graduation to K08, K23, or K99 award by degree type, as indicated for US medical school graduates.

Given that non-US medical graduates took significantly longer to receive CDAs, we further evaluated the effect of degree type and sex on time from medical school graduation to CDA among US medical school graduates (Figure 3). The median time from medical school graduation to K award was significantly less for MD-PhD graduates (8.3 years; range, 5.2–15.1 years) than for MD-only degree holders (9.3 years; range, 4.2–16.8 years, *P* = 0.002). Removing the 3 US MD-PhD holders who received their PhD after medical school from the MD-PhD group did not substantially change the median time to K award for MD-PhD degree holders (8.1 years; range, 5.2–13 years). There were no statistically significant differences in the time from medical school graduation to K award for women compared with men. For MD-PhD graduates, the median time from medical school graduation to K award was 8.1 years (range, 5.6–15.1 years) for men and 8.7 years (range, 5.2–14 years) for women. For MD-only degree holders the median time was 9.2 years (range, 6.0–15.2 years) for men and 9.7 years (range, 4.2–16.8 years) for women.

As nearly all of the US MD-PhD holders with K awards were K08 awardees, it was possible to meaningfully compare the time only to K08 award among graduates of US medical schools by degree types. Of the K08 awards to US medical school graduates, 33% (*n* = 25 of 75) were MD-only degree holders and 67% (*n* = 50 of 75) were MD-PhD degree holders. Those with MD-PhD degrees had a median time from medical school graduation to K08 award of 8.3 years (range, 5.2–15.1 years). This was significantly shorter than for MD-only degree holders who had a median time of 9.4 years (range, 7.0–15.1 years; *P* = 0.015).

For IK2 awardees who graduated from US medical schools, the median time from medical school graduation to IK2 was 6.8 years (range, 5.9–8.3 years) for MD-PhD graduates and 10.7 years (range, 9.3–12.8 years) for the combined MD-only and DO-only group.

## Discussion

Our results highlight some important factors relevant to the training of physician-scientists. Of MD-PhD graduates trained in internal medicine who received a K award, 92% received a K08 award; of MD-only graduates who received a K award, a majority received a K23 award. Individuals with MD-PhD degrees who graduated from US medical schools and completed internal medicine residency training took, on average, 8 years of postgraduate training before receiving a K award, and those without such an advanced degree took longer still. Interestingly, we found no sex differences in time from medical school graduation to K award. The time from medical school graduation to IK2 award was shorter than to K award for MD-PhD degree holders but not for MD-only degree holders. These data point to a critical dilemma for physician-scientists and the physician-scientist pipeline.

In 2022, the NIH reported that the median age of K applicants was 36–38 years (5). This analysis included people with MD, PhD, and MD-PhD degrees but did not break down age by K award type or the age at which the K application was awarded. By exploring data for graduates of internal medicine residency programs, we show that MD graduates who pursue training in internal medicine and its subspecialties are spending on average 8–10 years in postgraduate training prior to receiving a K award. If the average age at entry to medical school is 24 years (6), those individuals who aspire to research careers in internal medicine and its subspecialties will be approximately 36–40 years old at receipt of an NIH CDA. While those with MD-PhDs required 1 or 2 years less postgraduate training before receiving a K award, they also graduate from medical school at an older age compared with MD-only degree holders. Thus, any advantages to achieving a CDA afforded by an advanced degree are neutralized by the total training time required. For MD-PhD graduates, this may also translate into a shorter time to independent research funding and higher success rates with R01 or equivalent applications. This possibility is supported by the fact that, in 2020, the age to first R01 was 46 years for both MD and MD-PhD graduates, despite MD-PhDs typically being older than MDs at the time of medical school graduation (4). Interestingly, although the numbers were small, we found that the time to IK2 award was shorter than time to K award for MD-PhD graduates, but not MD-only graduates, which may reflect differences in how applications by MD-PhD graduates are reviewed by K and IK2 study sections.

From 1970 through the early 1990s, the average age at first R01 for MD-PhD graduates remained relatively stable at approximately 39 years. For MD-only graduates, it increased from 36 years in 1970 to 39 years in 1990 (7). Since then, the average age at first R01 has increased for both groups and reached a new steady state of 43–46 years (1). This finding is remarkable, and it is unclear why 4–7 additional years of training has become necessary for physician-scientists to achieve independence (4). This extended period of postgraduate training has significant implications for the future of the physician-scientist workforce and is an important factor in rates of both entry into and attrition from research careers. For one, academic institutions must support physician-scientists for extended periods to shepherd them to independence. Furthermore, prolonged training times have a major effect on the financial status of early-career investigators and their families. Finally, individuals who achieve independence in their mid-40s have a relatively short time span of research productivity until retirement.

At present, trainees typically spend 3 years in residency training after medical school graduation, followed by 2–4 years of fellowship training, some of which is devoted to research pursuits. Alternatively, residency training can be 2 years in the American Board of Internal Medicine (ABIM) Research Pathway. Subspecialty clinical fellowship training is then typically one to two years, and then up to 3 years protected research time. It is possible that more MD-PhD graduates pursued the ABIM Research Pathway than MD-only applicants, which shortened their time to K award. Overall, however, our data suggest that many trainees are receiving K awards after completing a number of years of postgraduate research training and as faculty rather than as fellows.

Our examination of the funded K and IK2 awards in 2022 revealed that the majority of awardees had MD degrees, rather than MD-PhD degrees. Indeed, in 2022 there were more MD-only applicants than MD-PhDs among K award applicants (5), and the same was true for all first independent research awards (e.g., R01) in 2020 (4). We also noted that almost all MD-PhD graduates received K08 awards, which typically fund more basic research, whereas those with MD-only degrees received K23 awards, which are associated with patient-oriented research (Figure 2). This distinction acknowledges that some NIH funding mechanisms may be more appropriate for physician-scientists who apply their clinical knowledge to basic discovery. In this regard, it is similarly important to not only support but also promote cohesion among a growing number of non-MD-PhD–focused physician-scientist training programs, such as the Physician-Scientist Support Foundation Medical Scholars Program, the Burroughs Wellcome Physician-Scientist Institutional Award Programs, the Doris Duke Charitable Foundation Clinical Scientist Development Award and Fund to Retain Clinical Scientist Programs, and the NIH R38 Stimulating Access to Research in Residency Programs.

The issue of time to first CDA should prompt serious thought about ways to shorten research training without affecting its quality. As medical schools are shortening the period of training and have veered away from the basic science curriculum of the past (8), it seems that for MDs who are considered “late bloomers” and become interested in research during their postgraduate clinical training, a longer period of research training in the postgraduate years would be important to gain equivalent research expertise to an MD-PhD graduate. An obvious and important consideration is that funding agencies should commit to supporting CDAs or first independent research grants at earlier career stages. This may include restructuring eligibility criteria and/or urging grant reviewers to appropriately balance promise and productivity. MD-PhD graduates who already have extensive research experience are often ready to move quickly to early independence awards (e.g., K99, DP5, or R01) rather than traditional K awards. Relative to all K awardees, we found that only 2% of internal medicine graduates received K99 awards in 2022, whereas among the total NIH K awardees (those receiving K08, K23, and K99s), 41% had K99s. This indicates that the vast majority of K99 awards are going to researchers with PhD degrees rather than physician-scientists.

An additional opportunity would be for academic institutions to develop their own programs to recognize and invest in promising physician-scientists to support them during their early career. This is fundamentally critical to help young physician-scientists to build momentum (9). Structured postgraduate research training through physician-scientist training programs could accelerate the time to K award and independent funding for physician-scientists. Finally, academic medical centers should create funding streams that support physician-scientists via novel approaches, most likely independent of traditional revenue streams.

It is important to recognize some important limitations of our study. First, this retrospective review relied on publicly available data, rather than data provided by grant awardees. However, the data sets generated from these sources are unbiased and, therefore, are entirely internally consistent. Second, we evaluated data from a single fiscal year (2022) of CDAs, and this year theoretically may not be representative of other years. It should be noted that the COVID-19 pandemic could also have had an effect on awardees during the time in which they were applying for their CDAs. As we used NIH RePORTER (https://reporter.nih.gov), we could only evaluate successful K and IK2 applications and, therefore, did not have information on unfunded applications or the number of submissions it took for successful applicants to obtain funding. We could not evaluate the proportion of K awardees who were from groups that are underrepresented in science and medicine, as this information is not available in NIH RePORTER or on institutional websites. Finally, at this point in time, we do not know how many of these K and IK2 awardees will obtain future R01 funding and whether shorter or longer latency to K award has any association with successful transition to independent funding or retention in academic research careers. It will take a few years to obtain these data from this cohort of K and IK2 awardees, but these will be important questions to investigate in the future.

In conclusion, training physician-scientists is critical to the future of biomedical research and clinical care. The length of this training process has increased to the point of being essentially unsustainable (3), likely contributing to the attrition of trainees, and ultimately limiting the duration of trainees’ career productivity. While prior studies have focused on the time to receive an MD-PhD degree, we found that the duration of time between postgraduate training and eventual federal CDA is equally as long, and we speculate that it is an impediment to effectively sustaining the physician-scientist pipeline. Major initiatives are needed to more rapidly propel physician-scientists to successful and independent research careers.

## Methods

To identify internal medicine residency graduates who received NIH K or VA IK2 awards, we searched NIH RePORTER for K01, K08, K23, K25, K99, and IK2 awards. K08, K23, and K99 awards were identified through the NIH RePORTER “advanced search” function between June and September 2023. Six separate searches were performed (1 for each activity type). Search criteria included: Fiscal Year 2022; Activity Code K01, K08, K23, K25, or K99 or IK2; and Support year 01. Subprojects were excluded. All fields from the search results were exported into Excel.

The data from the 5 K award searches were assessed for duplicate contact PIs, resulting in the identification of a total of 1,236 unique individual K awards (Supplemental Table 1). We excluded K01 awards, which are given to scientists without active clinical degrees. We individually evaluated the 13 K25 awardees (by online searches of the individual’s name and institution profile pages) and found that none were awarded to MDs, so these awards were also excluded, leaving 940 K08, K23, and K99 awards. These data were then merged in Excel (Figure 1). Of 940 K08, K23, or K99 awardees, 469 had their institutional department listed as “Internal Medicine/Medicine,” “Miscellaneous,” “None,” or “Unavailable” in NIH RePORTER, and were all included for further analysis. We then removed individuals with an institutional affiliation that was a known psychiatric or children’s hospital (*n* = 70) and those where the K review committee listed in NIH RePORTER was a nursing science or pediatric study section.

After exclusions, 388 awardees remained and underwent individual data abstraction through online searches of the Contact Principal Investigator (PI) Name and Organization Name. We examined online hospital and institution profile pages, professional social media profiles (e.g., Linkedin), and other websites that profile physicians (e.g., US News and World Report, Doximity) to find the complete information about awardee education and training, including year of graduation from medical school, location of medical school (US vs non-US), degrees obtained, whether the trainee graduated from a US MD-PhD or DO-PhD program, whether the trainee obtained a PhD before medical school or after medical school graduation, whether the trainee obtained other advanced degrees (e.g., Master’s degree), type of residency training (internal medicine or other), duration of residency training, and subspecialty training, if any. For the IK2 awards, 69 awardees were identified. The department was listed as “unavailable” for all IK2 awards, so manual data retrieval was performed for all of these awardees through online searches as described for the above K awards.

For both K and IK2 awardees, if the PI did not hold an MD, DO, or international equivalent degree (e.g., MBBS), they were excluded from further evaluation (*n* = 156 K awardees, *n* = 42 IK2 awardees). For those who had an MD, DO, or international equivalent degree, those who did not complete an internal medicine residency program (*n* = 52 K awardees, *n* = 8 IK2 awardees) or completed a combined internal medicine/pediatrics residency program (*n* = 4 K awardees, *n* = 0 IK2 awardees), were removed.

For those who completed residency in internal medicine (*n* = 176 K awardees, *n* = 19 IK2 awardees), we recorded whether individuals had MD-only (including those with a Master’s degree), DO-only, MD-PhD, or DO-PhD degrees; year of graduation from medical school; location of medical school (US or non-US); sex (if specified on institutional website or assigned based on pronouns used to describe the individual); and specialty or subspecialty if the PI had completed subspecialty training. Data were primarily retrieved from institutional websites; however, if not all of the data were available on the institutional website (i.e., year of graduation), then the data were retrieved from publicly available data on Linkedin, Doximity, and US News and World Report websites.

### Statistics.

Date of medical school graduation was set to June 30th on the year of graduation. Time from graduation to K award was calculated in months to the career development notice of award date, and the result was divided by 12 to calculate years. Data were analyzed using Excel pivot tables and SPSS version 29.0. Data are presented as frequency and percentage for categorical variables, and mean, median, standard deviation, and range for continuous variables. Continuous variables that were not normally distributed were compared with Mann-Whitney *U* test when comparing 2 groups and independent samples with Kruskal-Wallis test for more than 2 groups. A *P* value of less than 0.05 was considered statistically significant.

### Study approval.

The data collected in this study were from publicly available sources, so the research was not considered to be human subjects research and, thus, was not subject to Institutional Review Board approval.

### Data availability.

The data from NIH RePORTER are publicly available, as were the other data collected at the time the online searches were performed (June to August 2023), and they will be made available by the corresponding author upon request.

## Author contributions

EJG, PRC, BIK, JMV, OAA, CDK, RAB, KYR, PJH, CMI, CSW, and DCR were involved in conceptualizing the manuscript. EJG performed the NIH RePORTER and online data retrieval. EJG, DCR, and CSW prepared the first draft of the manuscript. EJG, PRC, BIK, JMV, OAA, CDK, RAB, KYR, PJH, CMI, CSW, and DCR reviewed the first draft, provided feedback, and edited the first and subsequent manuscript drafts.

## Supplementary Material

Supplemental data

## Figures and Tables

**Table 3:**
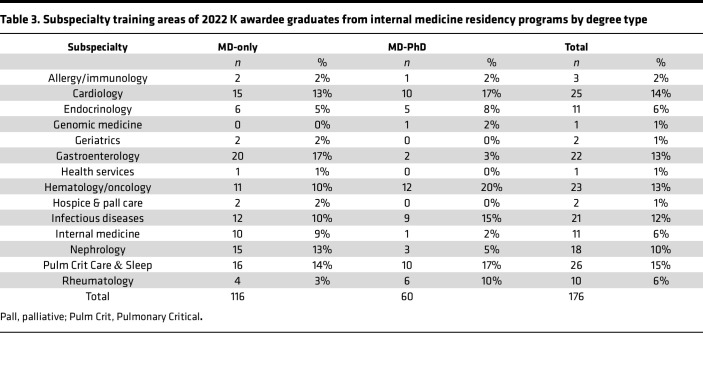
Subspecialty training areas of 2022 K awardee graduates from internal medicine residency programs by degree type

**Table 1:**
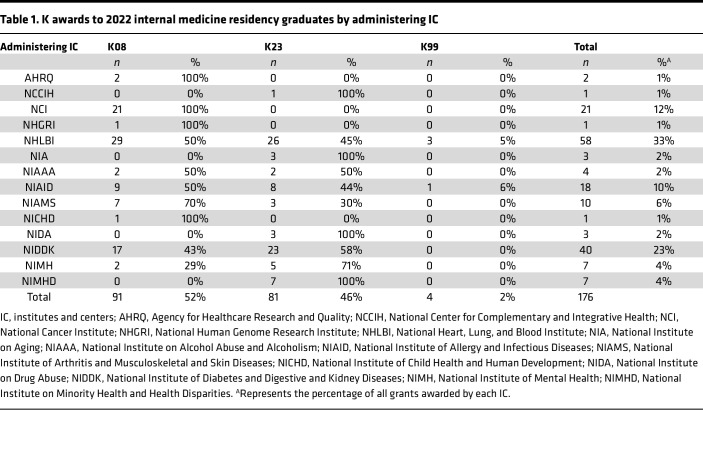
K awards to 2022 internal medicine residency graduates by administering IC

**Table 2:**
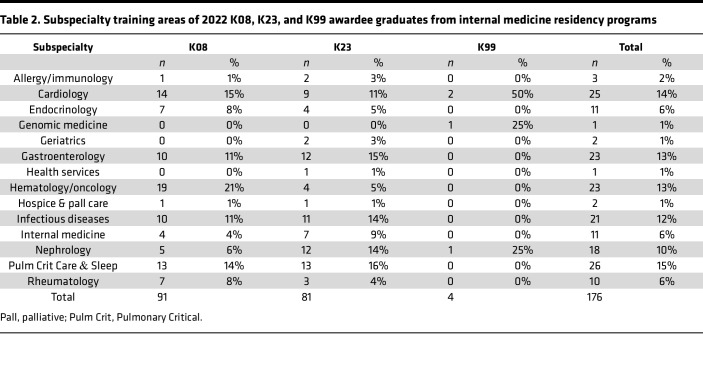
Subspecialty training areas of 2022 K08, K23, and K99 awardee graduates from internal medicine residency programs

## Version 1 05/22/2024

Electronic publication
