# Supplementary material for: Is it time to reduce the length of postgraduate training for physician-scientists in internal medicine?
Source: JCI Insight. 2024 May 22;9(10):e178214. doi: 10.1172/jci.insight.178214 (PMC11141926; doi:10.1172/jci.insight.178214)
Supplement: Supplemental data [file jciinsight-9-178214-s240.pdf]

Supplementary Table 1:
All K08, K23, K99 by Administering IC.

	K01		K08		K23		K25		K99		Total	
**Institute**	**n**	**%**	**n**	**%**	**n**	**%**	**n**	**%**	**n**	**%**	**n**	**%***
AHRQ	7	35%	13	65%	0	0%	0	0%	0	0%	20	2%
FIC	7	100%	0	0%	0	0%	0	0%	0	0%	7	1%
NCCIH	3	30%	1	10%	6	60%	0	0%	0	0%	10	1%
NCI	5	5%	61	56%	0	0%	0	0%	42	39%	108	9%
NCIPC	1	100%	0	0%	0	0%	0	0%	0	0%	1	0%
NEI	0	0%	7	28%	9	36%	0	0%	9	36%	25	2%
NHGRI	1	9%	1	9%	0	0%	0	0%	9	82%	11	1%
NHLBI	28	16%	40	23%	60	35%	2	1%	41	24%	171	14%
NIA	56	46%	3	2%	17	14%	3	2%	43	35%	122	10%
NIAAA	12	29%	8	19%	4	10%	1	2%	17	40%	42	3%
NIAID	11	16%	16	24%	18	26%	1	1%	22	32%	68	6%
NIAMS	7	15%	14	30%	12	26%	1	2%	12	26%	46	4%
NIBIB	1	9%	2	18%	1	9%	1	9%	6	55%	11	1%
NICHD	5	8%	5	8%	25	39%	1	2%	28	44%	64	5%
NIDA	30	48%	3	5%	18	29%	1	2%	10	16%	62	5%
NIDCD	4	22%	2	11%	5	28%	0	0%	7	39%	18	1%
NIDCR	3	18%	4	24%	0	0%	0	0%	10	59%	17	1%
NIDDK	28	24%	31	27%	43	37%	1	1%	12	10%	115	9%
NIEHS	4	24%	1	6%	0	0%	1	6%	11	65%	17	1%
NIGMS	0	0%	1	2%	3	6%	0	0%	46	92%	50	4%
NIMH	31	30%	8	8%	43	42%	0	0%	21	20%	103	8%
NIMHD	18	42%	3	7%	15	35%	0	0%	7	16%	43	3%
NINDS	8	10%	19	23%	25	31%	0	0%	29	36%	81	7%
NINR	3	23%	0	0%	9	69%	0	0%	1	8%	13	1%
NIOSH	2	100%	0	0%	0	0%	0	0%	0	0%	2	0%
NLM	0	0%	0	0%	0	0%	0	0%	1	100%	1	0%
OD	8	100%	0	0%	0	0%	0	0%	0	0%	8	1%
**Total**	**283**	**23%**	**243**	**20%**	**313**	**25%**	**13**	**1%**	**384**	**31%**	**1236**	**100%**

Supplementary Table 2:
All K08, K23, K99 by Department.

Department	K08		K23		K99		Total
	n	%	n	%	n	%	n
ANATOMY/CELL BIOLOGY	0	0%	0	0%	10	3%	10
ANESTHESIOLOGY	3	1%	5	2%	1	0%	9
BIOCHEMISTRY	1	0%	0	0%	20	5%	21
BIOLOGY	0	0%	0	0%	17	4%	17
BIOMEDICAL ENGINEERING	0	0%	0	0%	9	2%	9
BIOPHYSICS	0	0%	0	0%	2	1%	2
BIOSTATISTICS & OTHER MATH SCI	0	0%	1	0%	5	1%	6
CHEMISTRY	0	0%	0	0%	3	1%	3
DENTISTRY	3	1%	0	0%	8	2%	11
DERMATOLOGY	3	1%	2	1%	2	1%	7
EMERGENCY MEDICINE	5	2%	4	1%	1	0%	10
ENGINEERING (ALL TYPES)	0	0%	0	0%	3	1%	3
FAMILY MEDICINE	1	0%	5	2%	0	0%	6
GENETICS	0	0%	0	0%	11	3%	11
INTERNAL MEDICINE/MEDICINE	61	25%	65	21%	32	8%	158
MICROBIOLOGY/IMMUN/VIROLOGY	0	0%	0	0%	8	2%	8
MISCELLANEOUS	2	1%	2	1%	4	1%	8
NEUROLOGY	5	2%	15	5%	12	3%	32
NEUROSCIENCES	1	0%	2	1%	17	4%	20
NEUROSURGERY	0	0%	1	0%	2	1%	3
NONE	9	4%	25	8%	28	7%	62
OBSTETRICS & GYNECOLOGY	0	0%	5	2%	3	1%	8
OPHTHALMOLOGY	6	2%	6	2%	2	1%	14
ORTHOPEDICS	2	1%	0	0%	4	1%	6
OTHER BASIC SCIENCES	1	0%	1	0%	8	2%	10
OTHER CLINICAL SCIENCES	0	0%	1	0%	0	0%	1
OTHER HEALTH PROFESSIONS	1	0%	6	2%	4	1%	11
OTOLARYNGOLOGY	1	0%	1	0%	3	1%	5
PATHOLOGY	4	2%	0	0%	7	2%	11
PEDIATRICS	21	9%	34	11%	7	2%	62
PHARMACOLOGY	0	0%	3	1%	10	3%	13

PHYSICAL MEDICINE & REHAB	0	0%	3	1%	3	1%	6
PHYSIOLOGY	0	0%	0	0%	11	3%	11
PSYCHIATRY	4	2%	22	7%	16	4%	42
PSYCHOLOGY	3	1%	4	1%	16	4%	23
PUBLIC HEALTH & PREV MEDICINE	4	2%	6	2%	12	3%	22
RADIATION-DIAGNOSTIC/ONCOLOGY	6	2%	0	0%	4	1%	10
SOCIAL SCIENCES	0	0%	1	0%	2	1%	3
SURGERY	9	4%	5	2%	3	1%	17
Unavailable	82	34%	85	27%	74	19%	241
UROLOGY	3	1%	2	1%	0	0%	5
VETERINARY SCIENCES	2	1%	1	0%	0	0%	3
**Total**	**243**		**313**		**384**		**940**
